# Dental Pulp Stem Cell‐Derived Intracellular Vesicles Inhibit OSCC by Delivering PTEN to Suppress PI3K/AKT/mTOR Signalling Pathway

**DOI:** 10.1111/cpr.70248

**Published:** 2026-06-10

**Authors:** Yu Luo, Qiang Qin, Wenting She, Xiangying Wang, Xiqin Li, Chenxuan Shu, Ruohan Li, Ziwei Li, Dongjie Fu, Yan He, Qingsong Ye

**Affiliations:** ^1^ Center of Regenerative Medicine and Department of Stomatology Renmin Hospital of Wuhan University Wuhan China; ^2^ Institute of Regenerative and Translational Medicine, Tianyou Hospital Wuhan University of Science and Technology Wuhan China; ^3^ Department of Oral and Maxillofacial Surgery, Massachusetts General Hospital Harvard Medical School Boston Massachusetts USA

**Keywords:** DPSC‐IVs, OSCC, PI3K/AKT/mTOR signalling pathway, PINK1/parkin mediated mitophagy

## Abstract

Oral squamous cell carcinoma (OSCC) represents a globally predominant type of oral malignancy with escalating incidence, featuring aggressive biological behaviour, prominent metastatic potential and poor clinical outcomes. Emerging evidence positions dental pulp stem cell‐sourced intracellular vesicles (DPSC‐IVs) as novel therapeutic vectors in regenerative oncology, citing their low immunogenicity, favourable safety profile and ability to modulate tumour microenvironment. In this study, DPSC‐IVs significantly inhibited OSCC progression both in vitro and in vivo, suppressing tumour cell proliferation, invasion and colony formation while simultaneously promoting apoptosis. Notably, the antitumor effect of DPSC‐IVs was further enhanced by combining them with autophagy inhibitor 3‐methyladenine (3‐MA), which synergistically suppressed the PI3K/AKT/mTOR pathway and enhanced mitochondrial stress while suppressing residual cytoprotective autophagy. Mechanistically, DPSC‐IVs served as carriers of PTEN into OSCC cells, which in turn suppressed oncogenic PI3K/AKT signalling and induced excessive mitophagy. Taken together, this study indicated that DPSC‐IVs could suppress OSCC through dual mechanisms, highlighting their potential as a promising and clinically translatable therapeutic option with advantages in safety and scalable production.

## Graphical abstract

DPSC‐IVs suppressed PI3K/AKT/mTOR signalling pathway by delivering PTEN to tumour cells, which reduced the expression of Bcl‐2 and elevated that of Bax. Meanwhile, DPSC‐IVs activated PINK1/Parkin mediated mitophagy, which further raised ROS levels. These effects suppressed tumour cell growth, triggered tumour cell apoptosis and improved overall condition of tumour‐bearing mice.

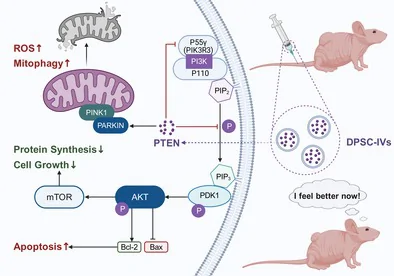

## 1 Introduction

Oral squamous cell carcinoma (OSCC) stands as one of the most prevalent oral malignancies globally, marked by aggressive behaviour, strong metastatic potential and unfavourable prognosis [1]. Over 300,000 new cases are diagnosed globally each year, yet 5‐year survival remains only around 50% [2, 3]. Despite incremental improvements in multimodal treatments, advanced OSCC still exhibits high rates of local recurrence and distant metastasis [4, 5]. These clinical challenges highlight the urgent need to develop biologically compatible therapeutic strategies for OSCC [6, 7].

Among the dysregulated oncogenic networks in OSCC, the PI3K/AKT/mTOR signalling axis represents a central regulator of tumour‐cell proliferation, survival, invasion, angiogenesis, metabolic reprogramming and treatment resistance [8, 9, 10]. Integrated molecular analyses have shown that abnormalities in this pathway occur in a substantial proportion of OSCC cases, mainly involving PIK3CA mutations, PTEN deficiency and AKT hyperphosphorylation [11, 12]. Clinically, aberrant activation of PI3K/AKT/mTOR signalling is closely associated with increased tumour invasiveness, lymph node metastasis and resistance to chemoradiotherapy [13, 14]. PTEN is the principal endogenous antagonist of the PI3K/AKT/mTOR pathway and suppresses downstream signal transduction by limiting PI3K‐dependent AKT activation [12, 14]. Therefore, PTEN loss, downregulation or functional insufficiency can sustain oncogenic PI3K/AKT/mTOR hyperactivation in OSCC [6]. Restoring PTEN activity is thus a conceptually attractive strategy for suppressing OSCC progression. However, efficient delivery of functional PTEN protein into tumour cells remains technically challenging [15].

Importantly, PI3K/AKT/mTOR signalling is not only a canonical proliferative and anti‐apoptotic pathway but also a major regulator of autophagy and mitochondrial quality control [16]. Mitochondria are essential regulators of cellular energy metabolism, redox balance and apoptosis, and dysregulated mitochondrial homeostasis has been increasingly implicated in OSCC tumorigenesis and progression [17]. Mitophagy, particularly Parkin/PINK1‐mediated mitophagy, selectively removes damaged mitochondria and preserves cellular homeostasis [18, 19]. However, the role of mitophagy in cancer is context‐dependent [20]. Basal or adaptive mitophagy may support tumour‐cell survival under hypoxia, nutrient deprivation or therapeutic stress, whereas excessive or dysregulated mitophagy can induce mitochondrial depolarisation, oxidative stress, mitochondrial collapse and intrinsic apoptosis [21, 22, 23]. Previous studies have also demonstrated that mitophagy interacts with PI3K/mTOR/NF‐κB‐related signalling networks and contributes to metabolic remodelling, stemness maintenance and therapeutic resistance in oral carcinoma [24, 25]. Therefore, therapeutic modulation of PI3K/AKT/mTOR signalling may not only suppress proliferation but also expose mitochondrial vulnerability by shifting mitophagy from a protective quality‐control towards a stress response [19, 20].

Vesicle‐based delivery systems provide a promising approach for transferring bioactive proteins, RNAs and lipids into target cells [26]. Stem cell‐derived extracellular vesicles (EVs) have attracted increasing attention because of their low immunogenicity, favourable biocompatibility and ability to mediate intercellular communication [27, 28]. Nevertheless, classical EVs are actively secreted by viable cells, and their translational application is often limited by low secretion yield, selective cargo sorting and batch‐to‐batch heterogeneity [29]. In this context, dental pulp stem cells (DPSCs) derived lysates appear to be attractive. Previous studies have reported that DPSC‐derived lysates can suppress lung cancer cell proliferation, migration, invasion and tumour growth, suggesting that intracellular components derived from DPSCs may possess antitumor bioactivity [30]. However, the specific vesicular entities responsible for this activity and their functional cargo remain undefined. Recent research has found that DPSC lysates contain abundant vesicles with EV‐like biological activity, which are named dental pulp stem cell‐derived intracellular vesicles (DPSC‐IVs) and isolated from DPSC lysates by ultracentrifugation [31]. Unlike classical EVs which are actively secreted into the extracellular microenvironment, DPSC‐IVs are cell‐intrinsic vesicular structures released from intracellular compartments after cell lysis. This unique origin may allow DPSC‐IVs to preserve intracellular molecular cargo from parental DPSCs, including tumour‐suppressive proteins that are difficult to enrich through EV secretion pathways. More importantly, compared to DPSC‐EVs, DPSC‐IVs have significantly higher yields [31]. Therefore, DPSC‐IVs may represent a distinct and scalable vesicle‐based platform for delivering functional antitumor cargo.

In this study, it was hypothesised that DPSC‐IVs could function as a natural intracellular vesicle‐based PTEN delivery system for OSCC therapy. Specifically, it was proposed that DPSC‐IVs deliver PTEN into OSCC cells, thereby suppressing the PI3K/AKT/mTOR signalling pathway and converting mitochondrial quality control into excessive PINK1/Parkin‐dependent mitophagy, ultimately inducing mitochondrial dysfunction, promoting apoptosis and inhibiting OSCC progression. To test this hypothesis, DPSC‐IVs were characterised and evaluated for their antitumor effects in OSCC models both in vitro and in vivo, with a particular focus on PTEN delivery, PI3K/AKT/mTOR pathway inhibition and Parkin/PINK1‐mediated mitophagy.

## 2 Materials and Methods

### 2.1 OSCC Cells Cultivation

The OSCC cell lines SCC25 and Cal27, provided by the School of Stomatology at Wuhan University, underwent propagation in glucose‐optimised DMEM (Sigma, USA) supplemented with 10% exosome‐depleted FBS (Gibco, USA) and a triple antibiotic/antimycotic formulation (Servicebio, China). Cultures were maintained within CO_2_/O_2_ modulated chambers.

### 2.2 Isolation and Culture of DPSCs

Third molars were obtained from healthy donors aged 12–25 years. The acquisition of human dental specimens was approved by the Laboratory Animal Ethics Committee of Renmin Hospital of Wuhan University (Approval No.: WDRY‐2022‐K025, Wuhan, China), and informed consent was secured prior to sample utilisation. Each extracted tooth was immediately immersed in PBS supplemented with penicillin–streptomycin (Gibco, USA). The supplement comprised 100 U/mL penicillin G and 100 μg/mL streptomycin (Servicebio, China). Dental pulp tissues were carefully dissected and minced into a homogeneous fine paste; they were then digested at 37°C for 1 h using a solution containing 4 mg/mL neutral protease (Roche, Switzerland) and 3 mg/mL type I collagenase (Sigma‐Aldrich, USA). After digestion, centrifugation of the samples was performed at 1000 rpm for 10 min. The obtained cell pellet was subjected to resuspension and cultured in α‐MEM (Gibco, USA) with 10% FBS (Gibco, USA) added to facilitate cell proliferation.

### 2.3 Preparation of DPSC‐IVs

To obtain DPSC‐IVs, a cell lysis‐based preparation strategy was adopted to avoid the overlap with EVs isolation from cell culture supernatant. DPSCs were trypsinised (Gibco, USA) into single‐cell suspension, which was subsequently washed three times with PBS to remove any residual EVs in the culture system. For every 1 × 10^7^ cells, subsequent to collection, the cell pellet was suspended in 1 mL of ultrapure water and incubated at 4°C for 30 min to achieve complete cell lysis. The cell suspension was placed in a −80°C freezer for 24 h and subsequently thawed at 4°C, with three repetitions of this freeze–thaw process. Following repeated freeze–thaw processing, the cell suspension underwent centrifugation at 4000*g* for 30 min in a centrifuge pre‐cooled to 4°C. The obtained supernatants were then purified by 2 cycles of ultracentrifugation in PBS (100,000*g*, 70 min) and the deposited DPSC‐IVs were re‐suspended in PBS.

### 2.4 In Vitro Tracing Experiment

SCC25 were trypsinised and gently pipetted to prepare single‐cell suspensions. Following quantification, a 6‐well plate was inoculated with 1000 cells per well. DPSC‐IVs were stained using 10 mM Dil, accompanied by a 30‐min incubation at 37°C in the dark. Subsequently, after three rinses with PBS, they were centrifuged at 7500*g* for 30 min via an Ultracel‐10 regenerated cellulose membrane (Millipore, USA) to remove unbound dye, prior to addition into the respective wells. For quantification, the fluorescence intensity of DPSC‐IVs internalised by SCC25 was assessed at 550 nm using a VICTOR Nivo multimode microplate reader (PerkinElmer EnSight, USA).

### 2.5 CCK‐8 Assay

3 × 10^4^ cells/mL SCC25 and Cal27 cells suspension were prepared, and each well of the 96‐well plate received 100 μL of the suspension, with six replicates per group. The surrounding wells were filled with an equal volume of PBS instead of cell suspension. A 37°C incubator was used to incubate the plate for roughly 4 h. Upon cell adherence to the well surfaces, 200 μL of DPSC‐IVs at concentrations of 0, 0.025, 0.035, 0.05 and 0.065 μg/μL was added to the matching wells. The CCK‐8 detection kit was brought to room temperature, with strict protection from light. Following removal of the culture medium, each well received 10 μL of CCK‐8 staining solution (MCE, USA) and 90 μL of serum‐free medium at each time point. The mixture was subjected to 2 h of incubation at 37°C, with subsequent measurement of absorbance at 450 nm via a microplate reader (PerkinElmer EnSight, USA).

### 2.6 Transwell Assay

After reaching 80% confluence in the logarithmic growth phase, SCC25 cells were rinsed three times with PBS, cultured in serum‐free medium for 24 h, prior to trypsinisation and counting. The upper chambers of 24‐well Transwell plates received cells at a density of 1 × 10^4^ per well in serum‐containing medium. After the cells attached, the upper chamber medium was switched to serum‐free medium. Meanwhile, the lower chambers were loaded with DPSC‐IVs at different concentrations (0, 0.025, 0.035, 0.05 and 0.065 μg/μL), with three replicate wells prepared for each concentration. After incubating for 24 h, the cells were subjected to three PBS washes, fixed in 4% paraformaldehyde and then underwent three more washes, stained with crystal violet and subjected to three additional washes. Cells remaining in the inner part of the upper chambers were wiped off. The membranes were imaged, and cells in four randomly selected fields of view were counted.

### 2.7 Cell Cloning Experiment

SCC25 and Cal27 were trypsinised and gently pipetted to prepare single‐cell suspensions. After cell quantification, 1000 cells per well were seeded into a 6‐well plate. DPSC‐IVs at different concentrations were added to respective wells, which were marked appropriately. The plate was maintained in static culture at 37°C with 5% CO_2_ in an incubator for 2 weeks, and the serum supplemented medium was renewed at 3 day intervals. Upon the formation of visible cell clones in the wells, cell culture was terminated. After three washes with PBS, cells were fixed in 4% PFA at room temperature for 15 min. Subsequent to one more wash, the cells were treated with a proper amount of crystal violet stain, followed by 15 min of incubation at room temperature. The plate was first rinsed with PBS to eliminate the staining solution completely, then air dried at room temperature prior to imaging.

### 2.8 Wound Healing Assay

SCC25 and Cal27 cells were inoculated into 6‐well culture plates at 5 × 10^5^ cells per well. Once the cells reached 90% confluency, a linear scratch was generated on the cell monolayer with a 200 μL pipette tip. Images of the scratch wounds were captured using a Leica light microscope (Germany).

### 2.9 Co‐Immunoprecipitation (Co‐IP) Assay

The cell culture medium in the plates was removed by aspiration, followed by two rinses of the cells with prechilled PBS. Subsequent experiments were performed using a Co‐IP kit (Beyotime, China). Initially, IP lysis buffer was supplemented to the cells, which was then followed by incubation on ice for 15 min to induce cell lysis. At 4°C, the cell lysate was centrifuged at 12,000 rpm for 5 min. Subsequently, 500 μg of protein from the supernatant had its final volume adjusted to 500 μL using prechilled PBS. Agarose Protein A + G beads were prepared for antibody conjugation and elimination of non‐specific binding. Prior to subsequent steps, the protein samples were combined with 50 μL of 50% agarose Protein A + G beads and incubated at 4°C, with gentle shaking maintained for 2 h. Subsequently, the sample was subjected to 5 min of centrifugation at 3000 rpm and 4°C to isolate the supernatant. Next, 3 μL of anti‐PIK3R3 antibody (Co‐IP antibody) or an equivalent amount of IgG (as control) was added to 500 μL of the total protein supernatant, and the mixture was gently shaken overnight at 4°C. Following the completion of antigen antibody binding, 50 μL of 50% agarose Protein A + G beads was supplemented, with incubation of the reaction at 4°C over 6 h. After collecting the beads through centrifugation at 3000 rpm for 5 min, they were rinsed three times using 1000 μL of pre‐cooled PBS each time and then resuspended in 120 μL of ×2 loading buffer. Following a 5 min boil, the resuspended mixture was kept on ice and subsequently subjected to centrifugation at 12,000 rpm for 5 min. Ultimately, 30 μL supernatant was loaded for Western blot analysis as previously described.

### 2.10 RNA Sequencing Analysis

After harvesting logarithmic phase SCC25, they were seeded into 6‐well plates and incubated in suitable medium. Two groups were set up in the experiment: the Cont group and the DPSC‐IVs group. Total RNA from cells of both groups was isolated with Trizol reagent, with subsequent library construction performed using the NEBNext Ultra RNA Library Prep Kit (NEB #E7490). Purification of the constructed libraries was performed using the AMPure XP system, aimed at removing impurities and ensuring the quality of the libraries. Finally, the Novaseq 6000 platform was employed for high throughput sequencing, with PE150 (paired‐end 150 bp) sequencing reagents applied to generate sequencing data supporting downstream gene expression analysis.

### 2.11 Bioinformatics Data Analysis

RNA sequencing data were analysed to identify gene expression differences between the Cont and DPSC‐IVs groups of SCC25 cells. Significantly differentially expressed genes (DEGs) were screened with the strict threshold of |log_2_fold change (FC)| ≥ 1 and false discovery rate (FDR) < 0.05. The obtained DEGs were further subjected to Gene Ontology (GO) enrichment analysis, Kyoto Encyclopedia of Genes and Genomes (KEGG) enrichment analysis, and cluster heatmap analysis to explore the underlying regulatory mechanism of DPSC‐IVs on SCC25 cells.

### 2.12 Western Blot

Samples of cells or tumour tissues were first collected, then lysed in RIPA buffer (Servicebio, China) containing a protease inhibitor cocktail (MCE, USA). The obtained homogenates were first centrifuged at 12,000*g* for 20 min at 4°C to eliminate insoluble precipitates. After centrifugation, the protein concentration in the supernatants was measured using a BCA assay kit (Beyotime, China). Protein samples of equal amounts were boiled in SDS loading buffer (Servicebio, China) to achieve denaturation. Following separation via 8%–12% Tris–gel SDS‐PAGE, protein transfer was performed onto a PVDF membrane (Servicebio, China). The membrane was first treated with TBST plus 5% non‐fat milk at room temperature for 2 h to block non‐specific antibody binding. Thereafter, primary antibodies against PI3K (Proteintech, China, 1:1000), p‐PI3K (Proteintech, China, 1:1000), AKT (Proteintech, China, 1:1000), p‐AKT (Proteintech, China, 1:1000), mTOR (Proteintech, China, 1:1000), p‐mTOR (Proteintech, China, 1:1000), Bax (Proteintech, China, 1:1000), Bcl2 (Proteintech, China, 1:1000), PINK1 (Proteintech, China, 1:1000), Parkin (Bioss, China, 1:1000), PTEN (Bioss, China, 1:1000), PIK3R3 (Bioss, China, 1:1000) and GAPDH (Servicebio, China, 1:5000) were employed to incubate at 4°C overnight, followed by 1 h of secondary antibody incubation at room temperature. An ECL chemiluminescent substrate was employed to visualise the protein bands. Ultimately, imaging was performed on the membrane using the ImageQuant 800 system (GE Healthcare, USA), followed by quantitative analysis of protein band grey values with ImageJ software.

### 2.13 Preparation of PTEN‐Knockdown DPSC‐IVs

To investigate whether PTEN carried by DPSC‐IVs contributed to the antitumor effects of DPSC‐IVs on OSCC cells, PTEN expression in DPSCs was knocked down using a lentivirus‐mediated short hairpin RNA strategy. DPSCs at passage 3 were seeded into six‐well plates and cultured until approximately 60%–70% confluence was reached. The cells were then transduced with lentiviral particles carrying PTEN‐specific shRNA, designated as the shPTEN group or a non‐targeting scrambled shRNA sequence, designated as the shNC group. Polybrene was added during transduction to enhance infection efficiency. After 24 h of incubation, the virus‐containing medium was replaced with fresh complete culture medium, and the cells were further cultured under standard conditions. Stable transduced DPSCs were selected using puromycin, and PTEN knockdown efficiency was verified by western blot analysis before subsequent experiments. DPSC‐IVs were isolated from shPTEN‐DPSCs and shNC‐DPSCs using the same DPSC‐IV preparation protocol and were referred to as IVs‐shPTEN and IVs‐shNC.

### 2.14 Nude Mouse Model Bearing SCC25

Male nude mice aged 4 weeks were acquired from the Laboratory Animal Center of Hubei Provincial Center for Disease Control and Prevention, with housing provided in the animal laboratory of Renmin Hospital of Wuhan University. The mice were given sterile water and feed freely, and acclimatised for 1 week in a standard environment maintained at 23°C–25°C room temperature, 55%–60% humidity and a 12 h light/dark cycle. The Laboratory Animal Ethics Committee of Renmin Hospital of Wuhan University granted approval for this animal experiment (Approval No.: WDRM20240302C). An equal volume of SCC25 cell suspension, prepared by resuspending 5 × 10^6^ cells in 200 μL serum‐free medium, was administered subcutaneously to each mouse, with drug administration starting 1 week after cell implantation. The control group (Cont) was intravenously injected with an equal volume of sterile phosphate‐buffered saline (PBS), while the experimental groups were given corresponding treatments of DPSC‐IVs and/or 3‐MA at the same injection volume and frequency. DPSC‐IVs were administered at a concentration of 1 μg/μL per mouse in 100 μL PBS by IV, IP or IT injection every 2 days for a 12‐day treatment. In the 3‐MA + DPSC‐IVs combination study, DPSC‐IVs, 3‐MA or their combined administration were performed intravenously with the same injection volume every 2 days for 20 days, during which DPSC‐IVs were delivered at 1 μg/μL per mouse in 100 μL PBS and 3‐MA was given at 1.5 mg per 100 g body weight in 100 μL PBS. Tumour growth was monitored throughout the experiment. After the drug treatment was finished, cervical dislocation was used to euthanise the mice. Tumour specimens were harvested, and vernier callipers were used to measure the maximum (*L*) and minimum (*W*) diameters of the tumours. The formula *V* = 0.5 × *L* × *W* ^2^ was applied to calculate tumour volume. The collected tumours were either fixed in 4% paraformaldehyde for subsequent paraffin embedding or preserved at −80°C for follow‐up experiments.

### 2.15 Histological and Immunofluorescence Analyses

After treatment with DPSC‐IVs, haematoxylin–eosin (H&E, Servicebio, China) staining and TUNEL (Servicebio, China) assay were conducted to assess tumour angiogenesis and the signalling pathways associated with OSCC. The expression levels of related indicators were determined via immunofluorescence staining, while cell nuclei were counterstained with DAPI (Servicebio, China) to facilitate visualisation. A microscope was utilised to capture microscopic images of the stained samples, while ImageJ software was applied to conduct image analysis and quantify the related experimental data.

### 2.16 Statistical Analysis

Each experiment underwent triplicate testing to ensure the reproducibility and accuracy of results, with statistical analyses implemented via GraphPad Prism 6.0 software. Specifically, Student's *t*‐test for two‐group differences and one‐way ANOVA for multi‐group comparisons were applied, with *p* < 0.05 indicating statistical significance.

## 3 Results

### 3.1 Characterisation of DPSC‐IVs

DPSC‐IVs, a cell‐intrinsic vesicle subtype distinct from classical actively secreted EVs, were extracted from DPSC lysates by ultracentrifugation (Figure S1A), and the isolated vesicles were subjected to comprehensive characterisation to verify their identity and distinguish them from EVs. DPSC showed a spindle‐like appearance under light microscope (Figure S1B). Alizarin Red S, Oil Red O and Alcian Blue staining results confirmed the multi‐lineage differentiation potential of DPSC (Figure S1C). The transmission electron microscope (TEM) showed that DPSC‐IVs were round shaped with a diameter below 200 nm (Figure S1D). Western blot results showed the presence of Alix, TSG101 and CD9 in DPSC‐IVs, while Calnexin was absent in DPSC‐IVs but present in DPSCs (Figure S1E). Nanoparticle tracking analysis (NTA) showed that the diameter of the DPSC‐IVs was below 200 nm (Figure S1F). In summary, these results confirmed that the collected vesicles were DPSC‐IVs.

### 3.2 DPSC‐IVs Inhibited OSCC In Vitro

SCC25 and Cal27 are classic OSCC cell lines with typical molecular characteristics of PI3K/AKT/mTOR pathway hyperactivation and PTEN low expression. The colony formation assay confirmed that DPSC‐IVs inhibited the proliferation of SCC25 and Cal27 in a concentration‐dependent manner (Figure 1A,B,G,H). Wound healing assays also showed DPSC‐IVs suppressed the cell migration of SCC25 and Cal27 (Figure 1C,D,I,J). Transwell assay revealed that the inhibitory effect of DPSC‐IVs on SCC25 invasion reached its maximum at a concentration of 50 μg/mL (Figure 1E,K). Flow cytometry results indicated DPSC‐IVs induced a significant dose‐dependent elevation in SCC25 cell apoptotic rate (Figure 1F,L). The CCK‐8 assay further confirmed the concentration‐dependent reduction in SCC25 and Cal27 viability treated with DPSC‐IVs (Figure 1M,N). These results indicated that DPSC‐IVs effectively inhibited the proliferation, migration, invasion and promoted apoptosis of the classical OSCC cell lines SCC25 and Cal27 in vitro.

**FIGURE 1:**
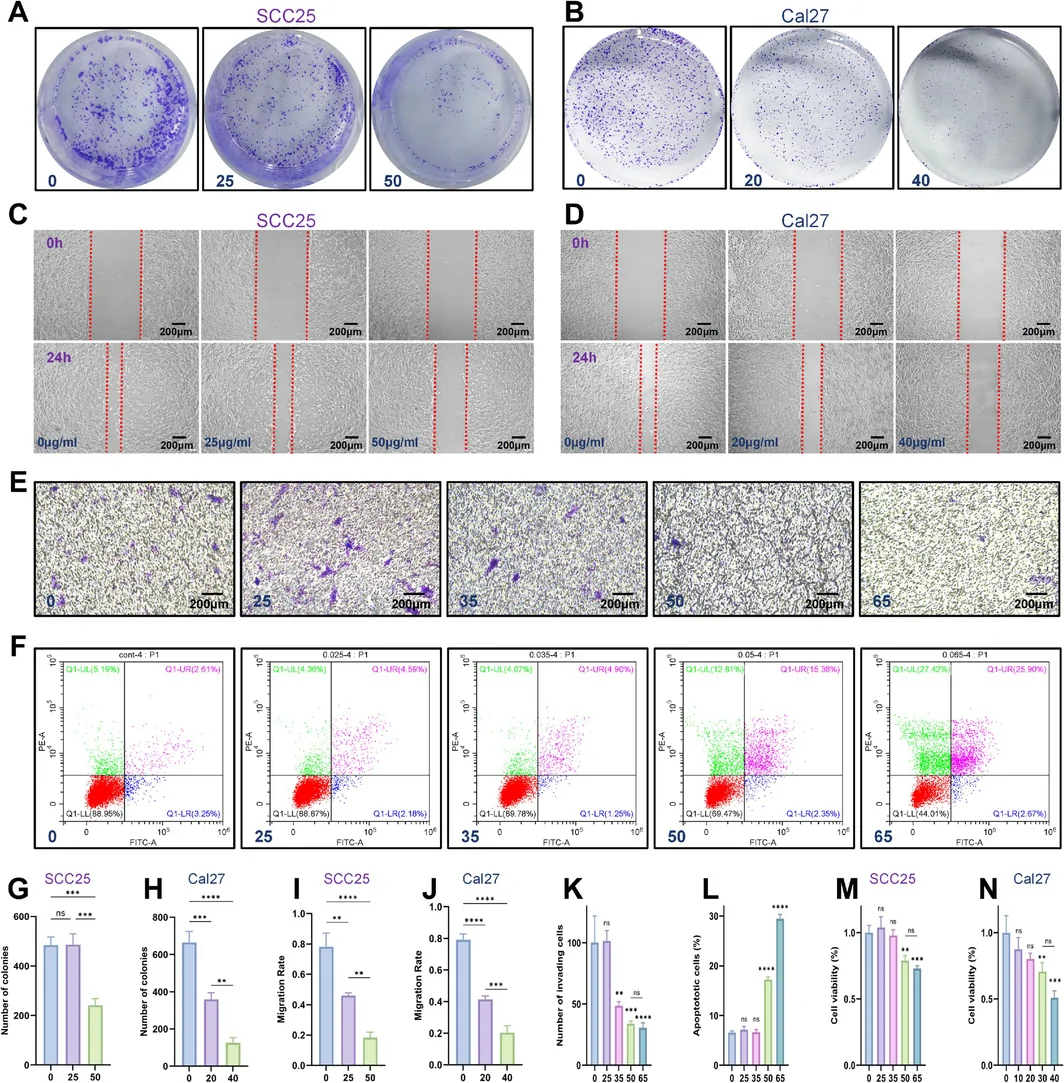
DPSC‐IVs inhibited SCC25 and Cal27 in a concentration‐dependent manner. (A) Colony formation assay showed DPSC‐IVs inhibited SCC25 colony formation in a concentration‐dependent manner, with concentrations of DPSC‐IVs = 0, 25, 50 μg/mL. (B) Colony formation assay showed DPSC‐IVs inhibited Cal27 colony formation in a concentration‐dependent manner, with concentrations of DPSC‐IVs = 0, 20, 40 μg/mL. (C) Wound healing experiment showed DPSC‐IVs = 50 μg/mL most effectively inhibited the migration of SCC25. Scale bar = 200 μm. (D) Wound healing experiment showed DPSC‐IVs = 40 μg/mL most effectively inhibited the migration of Cal27. Scale bar = 200 μm. (E) Transwell assay at DPSC‐IVs = 50 μg/mL showed optimal inhibition of SCC25 invasion. Scale bar = 200 μm. (F) Flow cytometry revealed concentration‐dependent increase in SCC25 apoptosis rate after DPSC‐IVs treatment. (G) Quantification of SCC25 colony formation from (A). (H) Quantification of Cal27 colony formation from (B). (I) Quantification of SCC25 migration rates in wound healing experiments from (C). (J) Quantification of Cal27 migration rates in wound healing experiments from (D). (K) Quantification of SCC25 invasion cells in transwell assays from (E). (L) Quantification of apoptotic SCC25 cells from (F). (M) CCK‐8 assay corroborated DPSC‐IVs induced concentration‐dependent viability decrease of SCC25, with concentrations of DPSC‐IVs = 0, 25, 35, 50, 65 μg/mL. (N) CCK‐8 assay corroborated DPSC‐IVs induced concentration‐dependent viability decrease of Cal27, with concentrations of DPSC‐IVs = 0, 25, 35, 50, 65 μg/mL (each group *n* = 3; values represented mean ± SD; statistical analysis: one‐way ANOVA with Tukey's multiple comparison test; ns indicates not significant, *p < 0.05, **p < 0.01, ***p < 0.001, ****p < 0.0001 compared to the Control group).

### 3.3 DPSC‐IVs Inhibited OSCC In Vivo

A SCC25 tumour‐bearing nude mouse model was constructed. Nude mice were given DPSC‐IVs via intravenous (IV), intraperitoneal (IP) or intratumoral (IT) injection and tumour responses were monitored among different groups (Figure 2A,B). The Cont and IT groups both displayed significant rises in tumour volume and weight, whereas those in the IV and IP groups were significantly reduced, indicating that IV or IP administration of DPSC‐IVs exerted significant antitumor effect (Figure 2C–F). Notably, there was no statistically significant difference in antitumor efficacy between IV and IP groups, suggesting both systemic administration routes effectively delivered DPSC‐IVs to OSCC xenografts and exerted inhibitory effects, while local IT injection failed to achieve the expected antitumor effect. Staining for Bax, Bcl2 and Ki‐67 showed that both IV and IP injections of DPSC‐IVs significantly promoted tumour cell apoptosis and inhibited malignant proliferation (Figure 2G–I). Immunofluorescence staining for Sox2 and N‐cadherin revealed that IV and IP injections of DPSC‐IVs effectively suppressed tumour cell invasiveness, with no statistically significant difference between the two routes of administration (Figure 2J,K). In contrast, no significant difference was observed in IT injection of DPSC‐IVs versus the Cont group. The above results indicated that IV or IP injection of DPSC‐IVs effectively inhibited tumour growth in vivo.

**FIGURE 2:**
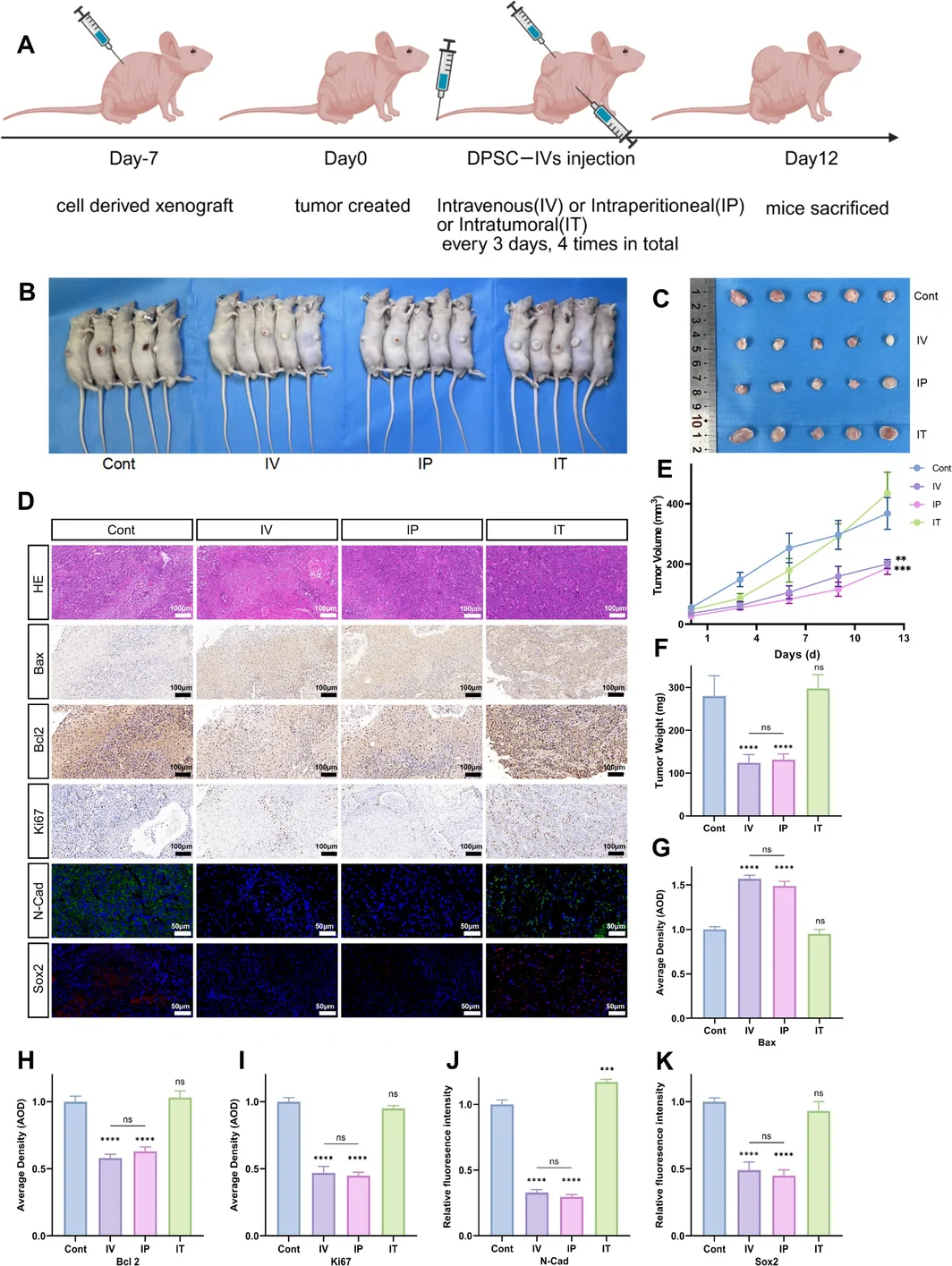
DPSC‐IVs inhibited OSCC in vivo (A and B) SCC25 tumour‐bearing nude mice were randomly divided into the control group (Cont, intravenously injected with an equal volume of sterile PBS) and experimental groups with DPSC‐IVs administered via intravenous (IV), intraperitoneal (IP) or intratumoral (IT) injection for a 12‐day treatment (*n* = 5). (C) After DPSC‐IVs IV or IP injection, tumour volumes and weights were significantly smaller, while the IT injection showed no significant difference compared to the Cont group (*n* = 5). (D) H&E staining, immunohistochemical (IHC) staining of Bax, Bcl2 and Ki‐67, scale bar = 100 μm; immunofluorescence (IF) staining of Sox2 and N‐cadherin in tumour tissue sections, scale bar = 50 μm. (E) Quantification of tumour volumes during 12‐day treatment (*n* = 5). (F) Quantification of tumour weights in Day 12 (*n* = 5). (G–K) Quantification of Bax, Bcl2, Ki‐67, Sox2 and N‐cadherin levels from (D) (*n* = 3). (*n* = 5 for in vivo groups, *n* = 3 for molecular detection; values represented mean ± SD; statistical analysis: one‐way ANOVA with Tukey's multiple comparison test; ns indicates not significant, **p < 0.01, ***p < 0.001, ****p < 0.0001 compared to the Control group).

### 3.4 DPSC‐IVs Inhibited OSCC by Suppressing PI3K/AKT/mTOR Axis

RNA sequencing was employed to compare transcriptomic differences of SCC25 between Cont group and DPSC‐IVs group, identifying 335 significantly downregulated and 889 significantly upregulated genes (screening threshold: |log_2_FC| ≥ 1, FDR < 0.05) (Figure 3A). These differentially expressed genes were mainly clustered in tumour‐related signalling pathways via KEGG enrichment analysis, with the PI3K/AKT pathway showing the highest enrichment (Figure 3B). Further analysis via cluster heatmap revealed that the Cont group expressed higher *PIK3R3* and *MTOR* than the DPSC‐IVs group, which are key genes of the PI3K/AKT/mTOR signalling pathway (Figure 3C). To further verify that DPSC‐IVs inhibited OSCC by suppressing the PI3K/AKT/mTOR signalling pathway, a western blot was performed in SCC25 and Cal27 cells. In both SCC25 and Cal27 cells, DPSC‐IVs treatment notably decreased p‐PI3K, p‐AKT and p‐mTOR expression compared with the Cont group. DPSC‐IVs also regulated apoptosis‐related proteins, with a specific decrease in the antiapoptotic Bcl2 and an increase in the proapoptotic Bax (Figure 3D–G). In conclusion, it could be inferred that DPSC‐IVs inhibited the phosphorylation of the PI3K/AKT/mTOR signalling pathway and increased the Bax/Bcl2 ratio, thereby inhibiting tumour proliferation and promoting apoptosis.

**FIGURE 3:**
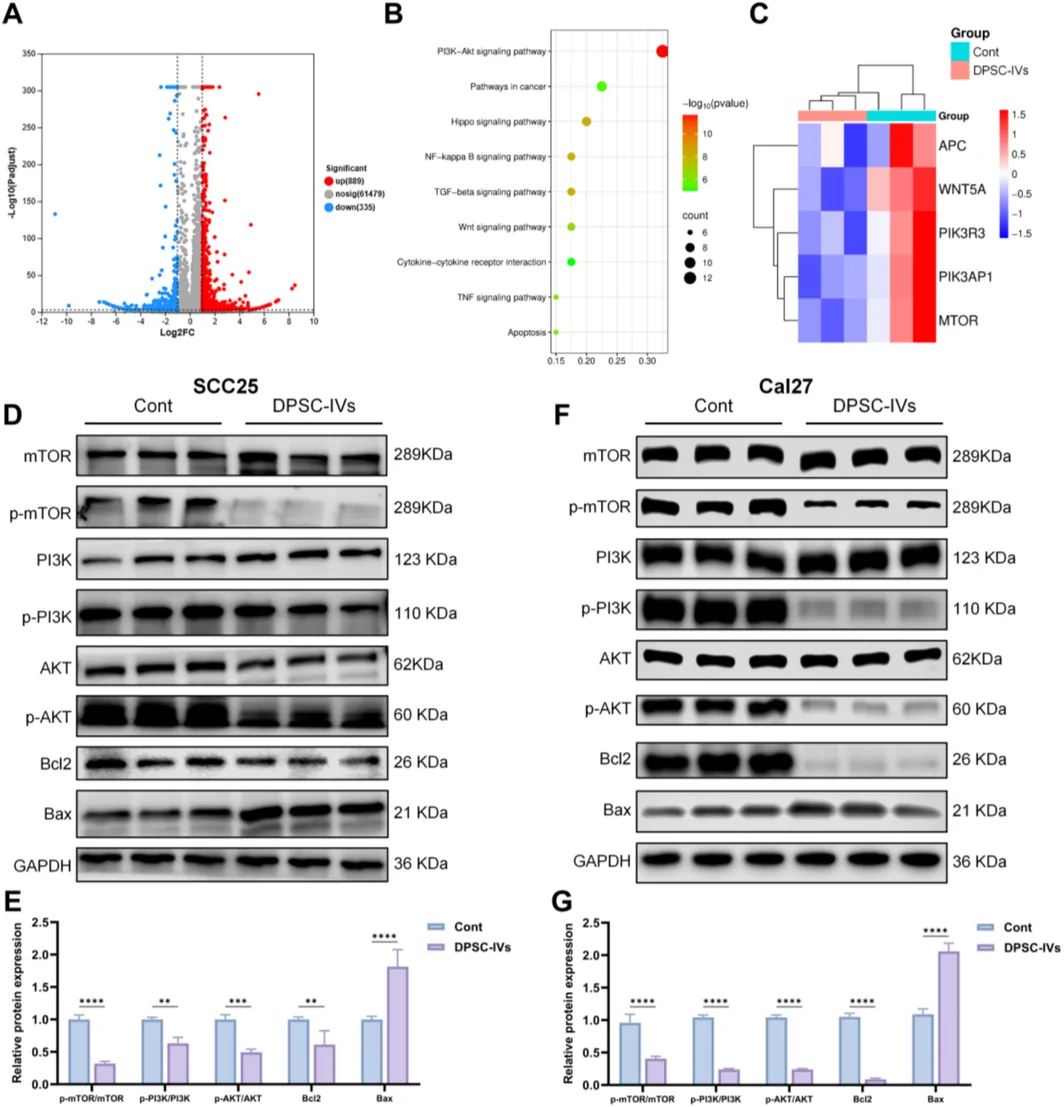
DPSC‐IVs inhibited OSCC by suppressing PI3K/AKT/mTOR signalling pathway. (A) RNA sequencing of SCC25 (Cont vs. DPSC‐IVs group) identified 335 downregulated and 889 upregulated genes (screening threshold: |log_2_FC| ≥ 1, FDR < 0.05). (B) KEGG enrichment analysis differentially expressed genes mainly enriched in the PI3K/AKT pathway. (C) Cluster heatmap highlighted PI3K/AKT/mTOR signalling pathway key genes *PIK3R3* and *MTOR* low expression in the DPSC‐IVs group compared to the Cont group. (D) Western blot of reduced p‐PI3K, p‐AKT, p‐mTOR, Bcl2 and increased Bax in SCC25 after DPSC‐IVs treatment. (E) Quantification of protein expression levels from (D). (F) Western blot of reduced p‐PI3K, p‐AKT, p‐mTOR, Bcl2 and increased Bax in Cal27 after DPSC‐IVs treatment. (G) Quantification of protein expression levels from (F) (each group *n* = 3; values represented mean ± SD; statistical analysis: Two‐group comparison used unpaired two‐tailed Student's *t*‐test; **p < 0.01, ***p < 0.001, ****p < 0.0001 compared to the Control group).

### 3.5 DPSC‐IVs Inhibited PI3K/AKT/mTOR Axis by Delivering PTEN

Given that PTEN acts as the most important key negative modulator of the PI3K/AKT/mTOR pathway, primarily impeding downstream signal transmission by suppressing AKT phosphorylation [32], it was supposed that the PI3K/AKT/mTOR pathway was inhibited by DPSC‐IVs via a PTEN modulatory approach. Fluorescence images further validated the efficient internalisation of DPSC‐IVs by SCC25 cells (Figure 4A), which demonstrated the active components of DPSC‐IVs could be taken up by tumour cells. Meanwhile, western blot confirmed the presence of PTEN protein in DPSC‐IVs, suggesting that DPSC‐IVs might suppress the PI3K/AKT/mTOR axis of SCC25 by delivering PTEN (Figure 4B). To verify whether DPSC‐IVs inhibited tumours by delivering PTEN, a PTEN knock‐down DPSC model was constructed. DPSCs were infected with lentivirus carrying PTEN‐specific shRNA (shPTEN) or scrambled control shRNA (shNC) to establish stable cell lines. Then the IVs derived from normal DPSCs, shNC or shPTEN‐infected DPSCs were used to treat SCC25. Western blot analysis revealed that the IVs derived from DPSCs significantly inhibited the phosphorylation of PI3K/AKT/mTOR and increased Bax/Bcl2. However, these effects were partially reversed after knocking down the PTEN of DPSCs (Figure 4C,D). DPSC‐IVs achieved this by delivering PTEN to inhibit the PI3K/AKT/mTOR signalling pathway and promote tumour cell apoptosis. Moreover, inhibitors and agonists of PTEN were used in SCC25 to further validate this conclusion. Treatment with PTEN agonist cefminox sodium further elevated PTEN expression, reinforced the effects of DPSC‐IVs which suppressed the PI3K/AKT/mTOR pathway and facilitated cell apoptosis. In contrast, the PTEN inhibitor VO‐Ohpictrihydrate partially blocked the effects of DPSC‐IVs (Figure 4E,F). Collectively, these findings verified that DPSC‐IVs exerted antitumor activity by delivering PTEN to inhibit the PI3K/AKT/mTOR signalling axis.

**FIGURE 4:**
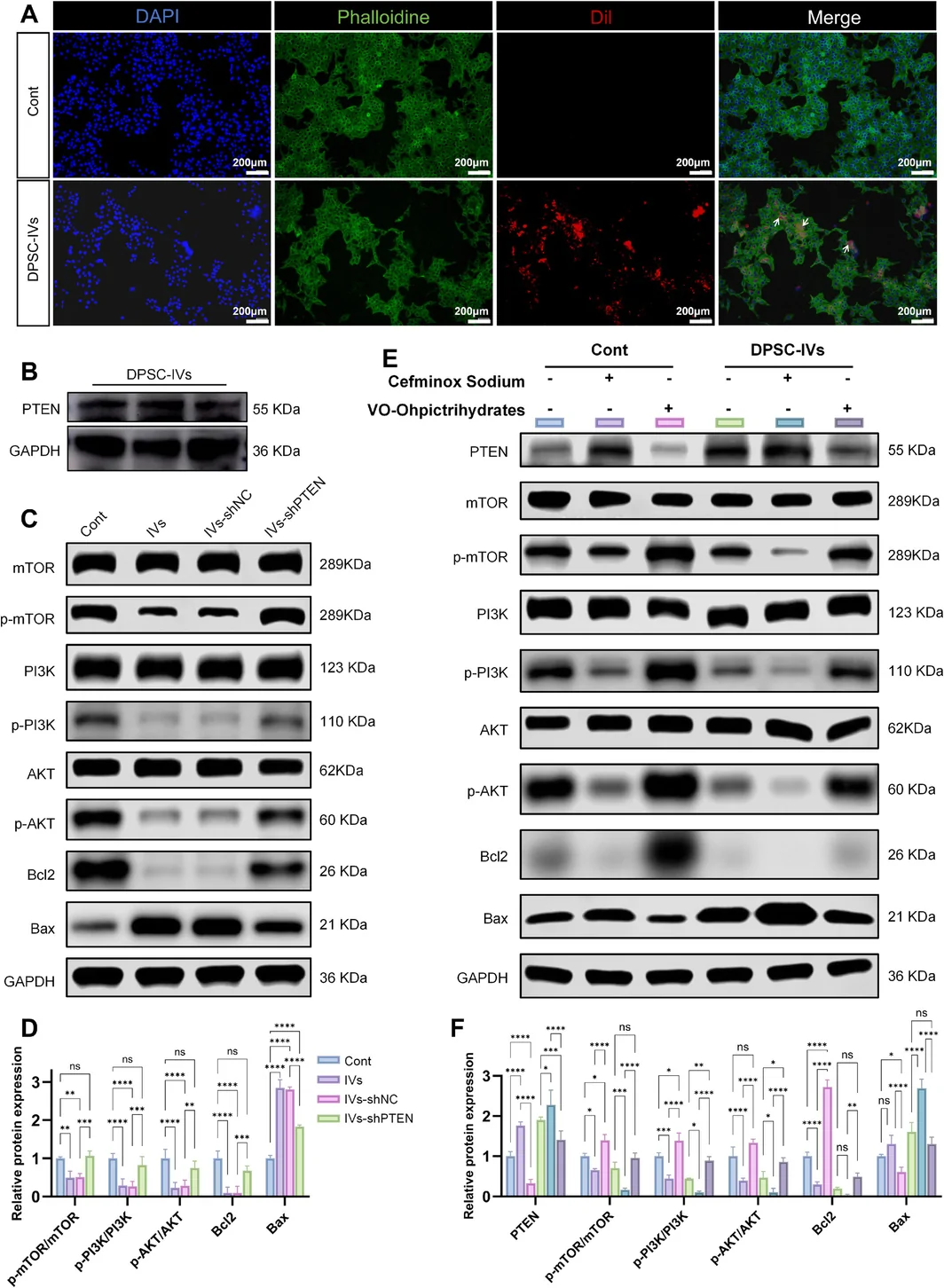
DPSC‐IVs inhibited PI3K/AKT/mTOR signalling pathway by delivering PTEN. (A) Fluorescence imaging confirmed efficient internalisation of DPSC‐IVs by SCC25 (as indicated by the white arrow). Scale bar = 200 μm. (B) Western blot indicated the presence of PTEN in DPSC‐IVs, the core negative regulator of PI3K/AKT/mTOR pathway. (C) Western blot of SCC25 treated with: PBS (Cont group), DPSC‐IVs (IVs group), IVs from DPSCs infected with lentivirus carrying scrambled control shRNA (IVs‐shNC group), IVs from DPSCs infected with lentivirus carrying PTEN‐specific shRNA (IVs‐shPTEN group). (D) Quantification of protein expression levels from (C). (E) Western blot of SCC25 treatment with DPSC‐IVs, PTEN agonist cefminox sodium or PTEN inhibitor VO‐Ohpictrihydrate. (F) Quantification of protein expression levels from (E) (each group *n* = 3; values represented mean ± SD; statistical analysis: multi‐group comparison used one‐way ANOVA with Tukey's multiple comparison test; ns indicates not significant, *p < 0.05, **p < 0.01, ***p < 0.001, ****p < 0.0001).

### 3.6 DPSC‐IVs Inhibited OSCC by Activating PINK1/Parkin‐Dependent Mitophagy

Predictions from the STRING database indicated direct interactions between PTEN, PIK3R3 and Parkin (Figure 5A), which was validated by Co‐IP assay in DPSC‐IVs‐treated SCC25 (Figure 5C). Further analysis of RNA sequencing data revealed that DPSC‐IVs treatment upregulated the expression of mitochondrial respiratory chain subunits (NDUFA1, NDUFC1, COX7C, COX17) (Figure 5B), suggesting that DPSC‐IVs may activate PINK1/Parkin‐mediated mitophagy through the respiratory chain overload induced oxidative stress axis. Examination by transmission electron microscopy demonstrated that the mitochondrial cristae of SCC25 in the Cont group maintained an intact structure, whereas those in the DPSC‐IVs group displayed significant mitochondrial damage (Figure 5D). JC‐1 assay findings revealed that compared with the Cont group, the red‐to‐green fluorescence intensity ratio in the DPSC‐IVs group was significantly lower (Figure 5E). Immunofluorescence staining revealed that the mitochondrial protein Tom20 (red) and PINK1 (green) colocalised. Specifically, DPSC‐IVs treatment induced the mitochondrial recruitment of Parkin, as verified by fluorescence colocalisation imaging. In contrast, Parkin was distributed across the entire cell in the Cont group (Figure 5F).

**FIGURE 5:**
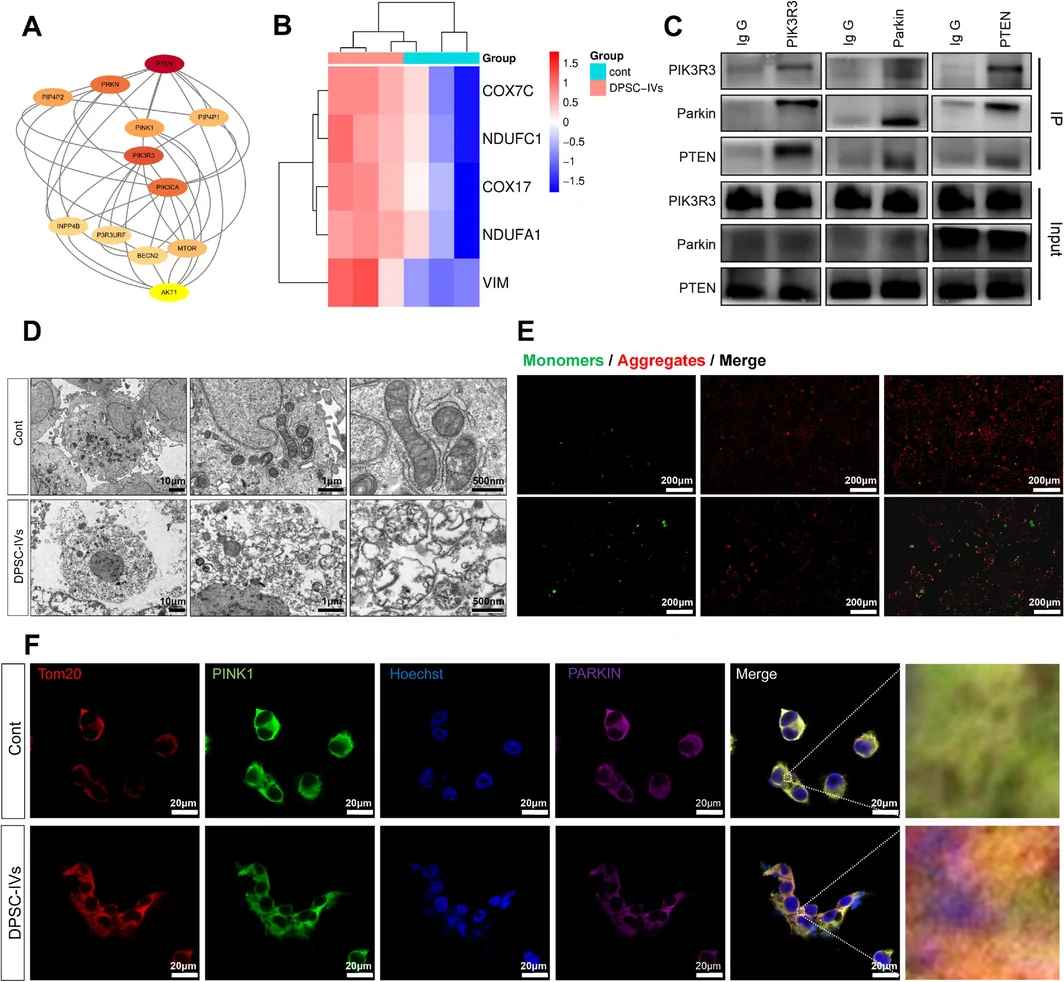
DPSC‐IVs activated PINK1/Parkin‐dependent mitophagy. (A) STRING predicted protein interaction between PI3K/AKT/mTOR pathway and PINK1/Parkin autophagy. (B) Heatmap highlighted DPSC‐IVs upregulated mitochondrial respiratory chain subunits. (C) CoIP verified PTEN‐PIK3R3‐Parkin interaction in DPSC‐IVs‐treated SCC25. (D) Electron microscopy discovered intact mitochondrial cristae in Cont group, and significant damage in DPSC‐IVs group. Scale bars = 10, 1 and 500 nm. (E) JC‐1 assay revealed a lower red/green ratio in DPSC‐IVs group, indicating mitochondrial membrane potential depolarisation. Scale bar = 200 μm. (F) Co‐localisation of mitochondrial protein Tom20 and PINK1 was observed by confocal microscopy. Scale bar = 20 μm.

### 3.7 Autophagy Inhibitors Augmented the Antitumor Efficacy of DPSC‐IVs Through PI3K/Akt/mTOR Axis In Vivo

An SCC25 tumour‐bearing nude mouse model was constructed, and tumour responses to a 20‐day treatment were monitored (Figure 6A). Tumour volume and weight increased markedly in the Cont group, while those in the 3‐MA, DPSC‐IVs and combination (3‐MA + DPSC‐IVs) groups were significantly reduced, confirming the effective antitumor activity of DPSC‐IVs (Figure 6B–F). H&E staining showed no significant differences in the heart, liver, spleen, lung and kidney among different groups, indicating that DPSC‐IVs had great biocompatibility and biosafety (Figure S2).

**FIGURE 6:**
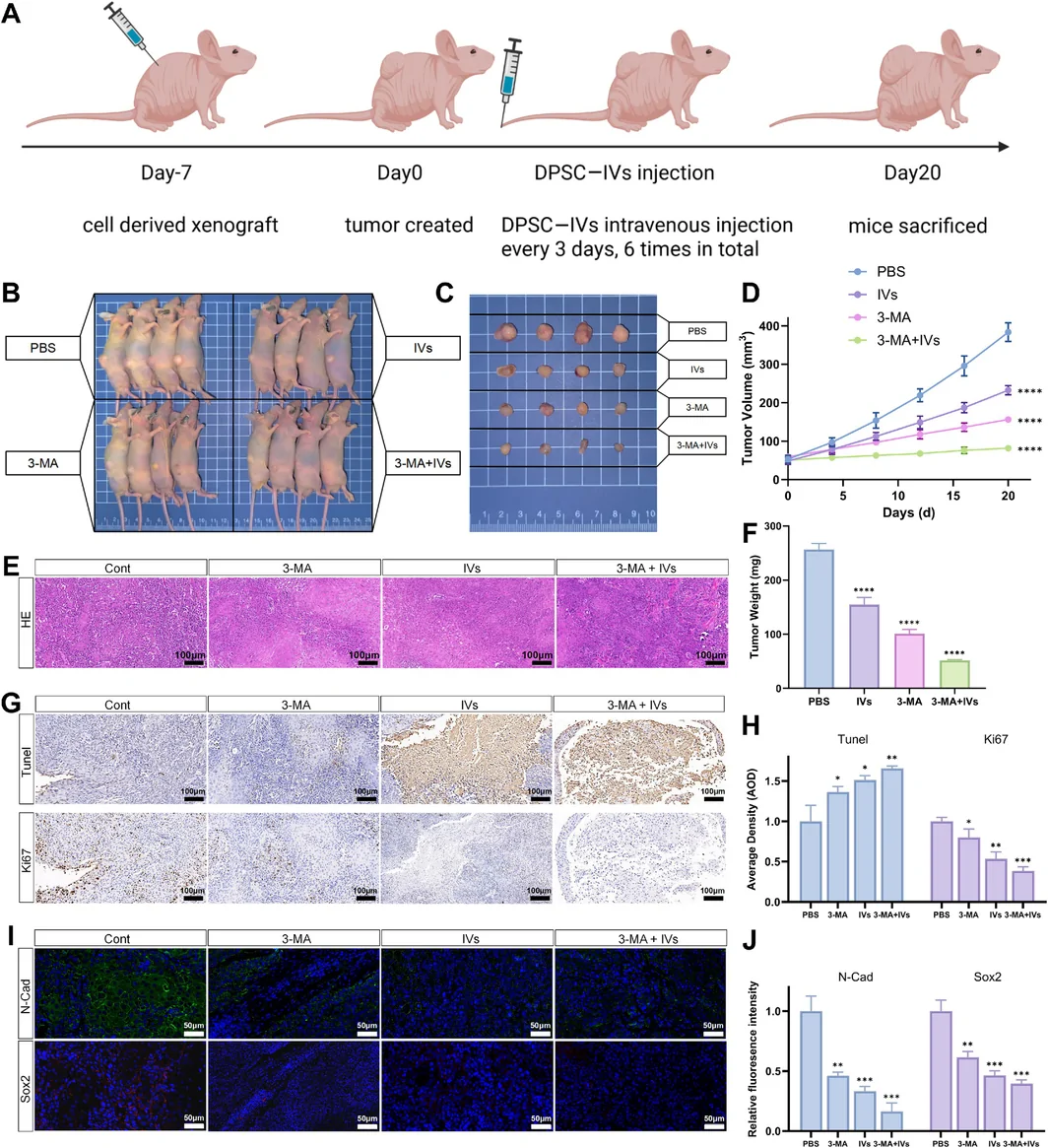
Autophagy inhibitors enhanced the antitumor effect of DPSC‐IVs in vivo. (A and B) SCC25 tumour‐bearing nude mice were randomly divided into the control group (Cont, intravenously injected with equal volume of sterile PBS) and experimental groups with 3‐MA, DPSC‐IVs or their combination administered via intravenous injection for a 20‐day treatment. (C) Autophagy inhibitors 3‐MA and DPSC‐IVs synergistically reduced tumour volumes and weights (*n* = 4). (D) Quantification of tumour volumes during 20‐day treatment (*n* = 4). (E) H&E staining in tumour tissue sections. Scale bar = 100 μm. (F) Quantification of tumour weights in Day 20 (*n* = 4). (G) Immunohistochemical (IHC) staining of Tunnel and Ki‐67 in tumour tissue sections. Scale bar = 100 μm. (H) Quantification of Tunnel and Ki‐67 levels from (G) (*n* = 3). (I) Immunofluorescence (IF) staining of N‐cadherin and Sox2 in tumour tissue sections. Scale bar = 50 μm. (J) Quantification of N‐cadherin and Sox2 levels from (I) (*n* = 3) (values represented mean ± SD; statistical analysis: One‐way ANOVA with Tukey's multiple comparison test; *p < 0.05, **p < 0.01, ***p < 0.001, ****p < 0.0001 compared to the PBS group).

Findings from TUNEL and Ki‐67 staining revealed that the 3‐MA, DPSC‐IVs and combination groups significantly induced tumour cell apoptosis and suppressed malignant proliferation (Figure 6G,H). The invasive and metastatic potential was further assessed by detecting Sox2 and N‐cadherin expression. Both proteins were downregulated in the 3‐MA, DPSC‐IVs and combination groups compared with the Cont group, indicating that DPSC‐IVs could inhibit the invasiveness of OSCC (Figure 6I,J).

IHC staining was used to evaluate PTEN expression and PI3K/AKT/mTOR signalling pathway protein phosphorylation in tumour tissues. The findings showed significant upregulation of PTEN expression in the 3‐MA, DPSC‐IVs and combined groups, along with significant inhibition of PI3K/AKT/mTOR phosphorylation levels. Notably, the combination group exhibited the most significant upregulation of PTEN and the strongest suppression of PI3K/AKT/mTOR phosphorylation (Figure 7A,B). These findings were further verified by Western blot analysis of tumour tissues (Figure 7C,D). Additionally, in the 3‐MA, DPSC‐IVs and combination groups, the ROS level in tumour tissues was significantly raised, among which the combination group showed the highest level (Figure 7E,F).

**FIGURE 7:**
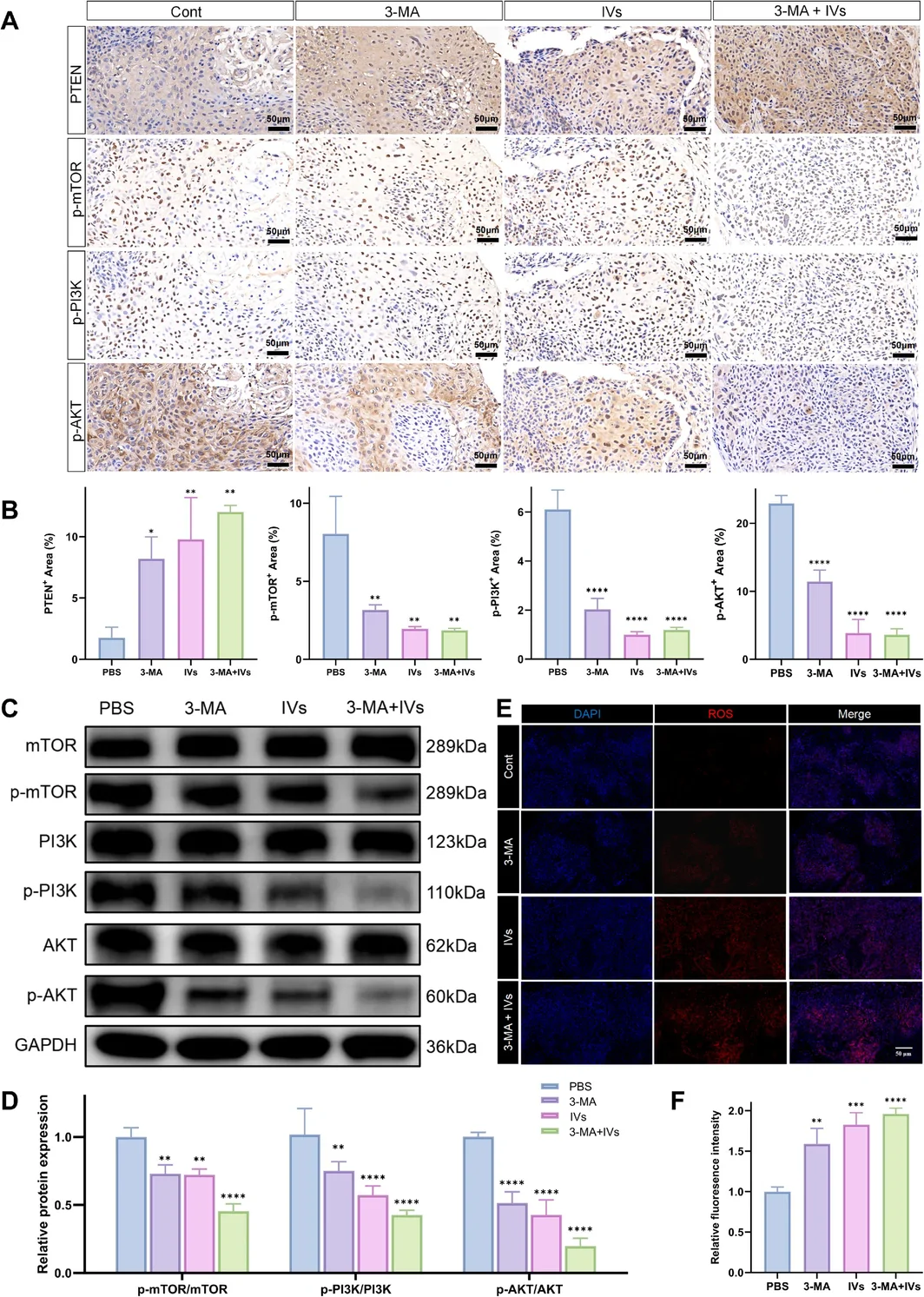
DPSC‐IVs inhibited OSCC in vivo via PI3K/AKT/mTOR signalling pathway. (A) Immunohistochemical (IHC) staining of PTEN and phosphorylation of PI3K/AKT/mTOR in tumour tissue sections. Scale bar = 50 μm. (B) Quantification of PTEN, p‐mTOR, p‐PI3K and p‐AKT levels from (A). (C) Western blot of reduced phosphorylation of PI3K/AKT/mTOR in tumour tissue after DPSC‐IVs and 3‐MA treatment. (D) Quantification of protein expression levels from (C). (E) Fluorescence images showed increased ROS in tumour tissue after DPSC‐IVs and 3‐MA treatment. Scale bar = 50 μm. (F) Quantification of ROS levels from (E) (each group *n* = 3; values represented mean ± SD; statistical analysis: one‐way ANOVA with Tukey's multiple comparison test; **p < 0.01, ***p < 0.001, ****p < 0.0001 compared to the PBS group).

In conclusion, 3‐MA, an autophagy inhibitor, could augment DPSC‐IVs' suppression of the PI3K/AKT/mTOR pathway, thereby potentiating their antitumor efficacy.

## 4 Discussion

This study demonstrated that DPSC‐IVs functioned as a cell‐free antitumor vesicle therapy for OSCC by transferring PTEN and thereby coupling PI3K/AKT/mTOR inhibition with PINK1/Parkin‐dependent mitochondrial collapse. The naturally derived intracellular vesicle subtype restored a tumour‐suppressive signal frequently attenuated in OSCC, while simultaneously converting mitochondrial quality control from an adaptive process into a pro‐apoptotic vulnerability (Scheme 1).

**SCHEME 1:**
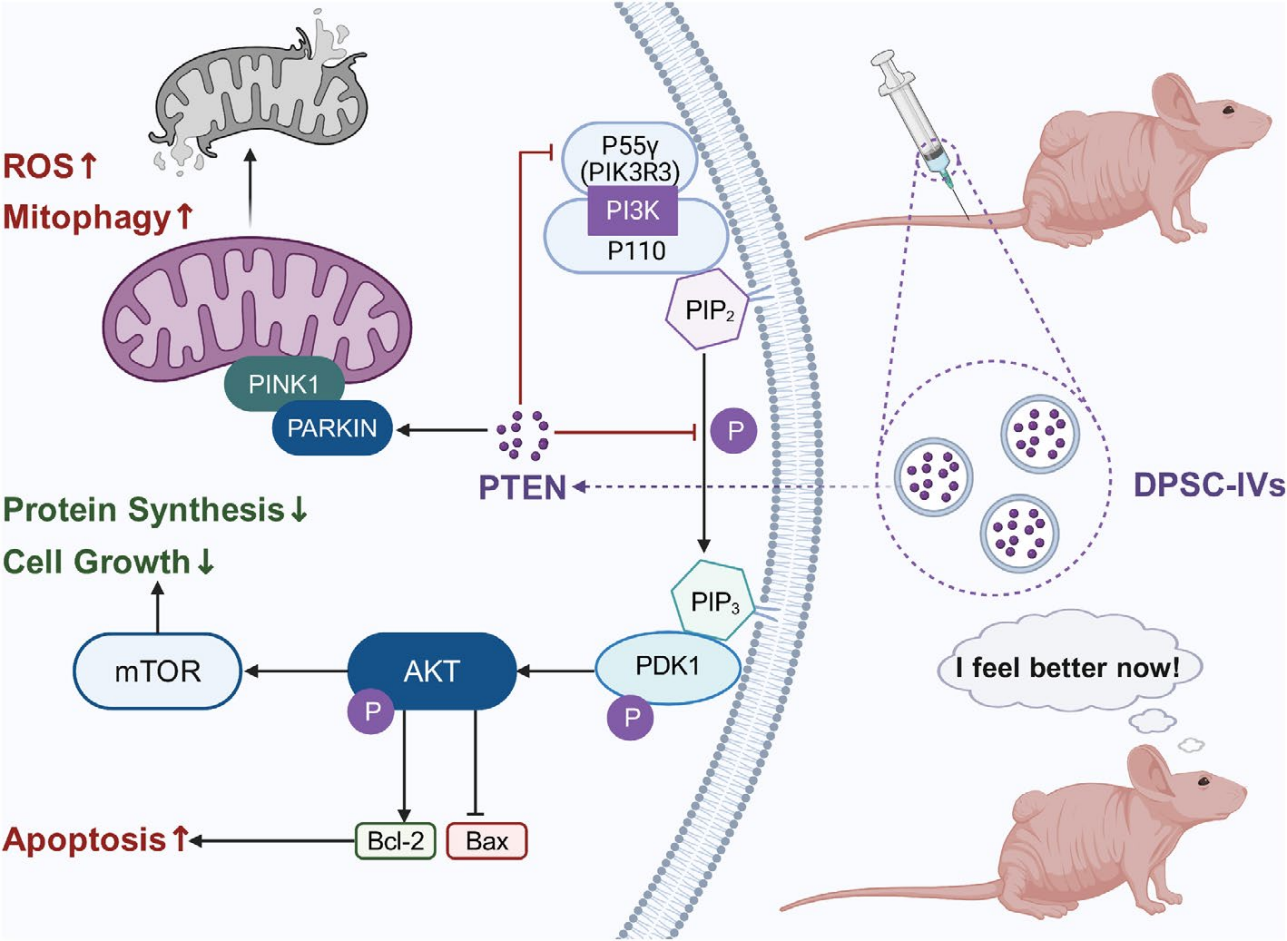
Schematic illustration of DPSC‐IVs inhibiting OSCC. DPSC‐IVs inhibited the PI3K/AKT/mTOR signalling pathway and enhanced PINK1/Parkin mediated mitophagy by delivering PTEN, which in turn increased Bax and decreased Bcl2 and ultimately promoted tumour apoptosis.

The antitumor phenotype observed in SCC25 and Cal27 cells was explained by restoring a PTEN‐dominant regulatory axis [6, 33]. OSCC remains clinically challenging because of recurrence, invasion and metastatic dissemination [34, 35]. At the molecular level, aberrant PI3K/AKT/mTOR signalling is a recurrent driver of OSCC progression, invasion, survival and therapeutic resistance, and PTEN loss or functional insufficiency is a key mechanism sustaining this pathway [14]. As the major endogenous phosphatase that specifically dephosphorylates PIP3, PTEN is the core negative regulatory molecule of the PI3K/AKT/mTOR pathway [12]. The detection of PTEN within DPSC‐IVs, efficient vesicle uptake by OSCC cells, reduced phosphorylation of PI3K, AKT and mTOR, and reciprocal regulation of Bax and Bcl2 provided a coherent mechanistic sequence. The IVs‐shPTEN and pharmacologic PTEN‐modulation experiments further strengthened causality: depletion of PTEN in parental DPSCs weakened the inhibitory effect of IVs, whereas PTEN activation reinforced pathway suppression. Moreover, the RNA‐seq results showed that the changes in the OSCC cell transcriptome after DPSC‐IVs treatment were almost all concentrated in the PI3K/AKT/mTOR pathway and its downstream apoptotic and proliferative signalling networks, which further confirmed that PTEN, as the core regulator of this pathway, was the key effector molecule mediating the antitumor effect of DPSC‐IVs.

A second mechanistic layer was the induction of mitochondrial stress and PINK1/Parkin‐dependent mitophagy. In DPSC‐IVs treated cells, hallmarks of PINK1/Parkin pathway activation were observed: loss of mitochondrial membrane potential, disrupted cristae, recruitment of Parkin to mitochondria, and upregulation of respiratory chain subunits [36]. Because mTOR is a central autophagy brake, PTEN‐driven mTOR inhibition may promote mitophagy‐related responses [16, 19]. Notably, the mitophagy induced by DPSC‐IVs was a PINK1/Parkin‐mediated, completely activated and excessive mitophagy, which is fundamentally different from the basal protective autophagy of OSCC cells: the basal autophagy is a low‐level cytoprotective process that clears mild cellular damage and maintains metabolic homeostasis under stress, and is mainly regulated by the classical PI3K/Beclin1/Vps34 axis; while DPSC‐IVs‐induced mitophagy was an over‐activated mitochondrial‐specific autophagy, triggered by PTEN‐mediated PI3K/AKT/mTOR pathway inhibition and subsequent oxidative stress [20, 36]. The DPSC‐IVs induced excessive mitophagy appeared to trigger a mitochondrial crisis that led to intrinsic apoptosis. Moreover, co‐administering the autophagy inhibitor 3‐MA further improved tumour control, indicating that residual autophagy could buffer stress and short‐term autophagy blockade collapsed this buffer, thereby increasing susceptibility to apoptosis [37, 38, 39]. Together, these results supported a dual hit model: PTEN delivery extinguished proliferative signalling and mitophagy overload pushed cells into committed apoptosis, in which PTEN acted as the initial and core trigger of the entire antitumor signal cascade.

The in vivo results added delivery route relevant insight. IT injections often only cover a small region of the tumour, leading to insufficient drug distribution and rapid systemic clearance [40]. High interstitial pressure and poor convective transport in solid tumours impede homogeneous intratumoral distribution of nanoscale agents, whereas systemic administration allows broader vascular access and predominantly perivascular uptake in tumours [41]. On this basis, IV administration was selected as the primary route for subsequent combination studies with 3‐MA, owing to its superior systemic bioavailability and more consistent drug exposure compared to IP delivery. In terms of pharmacokinetic differences, IV delivery bypasses the peritoneal absorption barrier, resulting in a faster onset of action and a more predictable plasma concentration of DPSC‐IVs, while IP delivery is associated with slower and passive diffusion into the bloodstream [42, 43, 44]. Clinically, IV delivery holds greater translational relevance for OSCC management, as OSCC often exhibits early lymph node metastasis and potential distant dissemination, and systemic delivery can address both primary tumours and micrometastases [34, 35]. Collectively, the pharmacokinetic and clinical translational merits of IV made it the optimal route for DPSC‐IVs therapeutic strategies.

Compared with existing vesicle‐based strategies, the novelty of DPSC‐IVs lay in cargo origin, yield logic and mechanism. Conventional DPSC‐derived exosomes have shown favourable regenerative and anti‐inflammatory activities, but actively secreted EVs are constrained by selective cargo sorting and relatively low production yield [45]. Artificial cell‐derived vesicles from DPSCs have recently been proposed to improve acquisition efficiency while maintaining EV‐like bioactivity [46]. In contrast to classical DPSC exosomes that rely on active secretion [27], DPSC‐IVs were isolated directly from native DPSC lysates without exogenous chemical or physical modification, preserving intracellular protein cargo such as PTEN. Furthermore, the preparation of DPSC‐IVs via freeze–thaw lysis and ultracentrifugation enables better batch consistency and scalable production, which is a critical advantage for clinical translation.

Academically, this study integrated PTEN delivered by IVs, oncogenic signalling PI3K/AKT/mTOR and excessive mitophagy into one mechanistic framework. It suggested that durable OSCC control may require both suppression of proliferative signalling and disruption of mitochondrial stress tolerance. Translationally, DPSC‐IVs offered a biocompatible vesicle system for PTEN‐deficient or PI3K/AKT‐hyperactive tumours. Future development should prioritise standardised GMP‐compatible production, cargo potency assays, biodistribution profiling, immune‐competent and patient‐derived models and rational pairing with clinically applicable autophagy inhibitors [47, 48].

The study has limitations. The in vivo work used immunodeficient xenografts and mainly SCC25 tumours, limiting evaluation of immune responses and intertumoral heterogeneity. In addition, 3‐MA is a mechanistic tool compound with limited specificity. Future studies should validate DPSC‐IVs in patient‐derived and immune‐competent OSCC models and test clinically relevant autophagy modulators.

In summary, PTEN‐enriched DPSC‐IVs suppressed OSCC by inhibiting PI3K/AKT/mTOR signalling and driving PINK1/Parkin‐dependent mitochondrial dysfunction towards apoptosis. These findings defined DPSC‐IVs as a promising cell‐free vesicle platform and supported further optimisation of systemic delivery, cargo standardisation and combination regimens for OSCC therapy.

## 5 Conclusion

In conclusion, this study demonstrated that DPSC‐IVs exerted potent antitumor effects against OSCC by delivering PTEN and suppressing PI3K/AKT/mTOR signalling. DPSC‐IVs inhibited proliferation, migration, invasion and tumour growth, while apoptosis was promoted both in vitro and in vivo. Mechanistically, PTEN‐containing DPSC‐IVs reduced PI3K/AKT/mTOR phosphorylation and induced PINK1/Parkin‐dependent mitochondrial dysfunction, shifting mitochondrial quality control towards pro‐apoptotic stress. The therapeutic efficacy of DPSC‐IVs was further enhanced by 3‐MA. These findings identified DPSC‐IVs as a scalable, biocompatible, cell‐free vesicle platform and supported their further development as a PTEN‐based therapeutic strategy for OSCC.

## Author Contributions

Q.Y. and Y.L. initiated the study concept. Q.Y. and Y.H. devised the experimental methods. The bulk of experiments and subsequent data analysis were executed by Y.L., Q.Q. and W.S. Y.L., Q.Q., X.W. and Z.L. gathered relevant data and conducted statistical assessments. Q.Y. and Y.H. orchestrated and supervised the entire experimental process. Y.L., Q.Q. and R.L. authored the manuscript. All authors have read through and endorsed the final version of the manuscript.

## Funding

This work was supported by the State Key Project Ministry of Science and Technology of China (2022YFC2504200) and the National Natural Science Foundation of China (82571057).

## Conflicts of Interest

The authors declare no conflicts of interest.

## Supporting information


**Figure S1:** Characterisation of DPSC‐derived intracellular vesicles (DPSC‐IVs). (A) Schematic illustration of the DPSC‐IVs extraction by ultracentrifugation. (B) Images of DPSCs‐P0 and DPSCs‐P3 under light microscope. Scale bar = 200 μm. (C) Alizarin red, oil red and alizarin blue staining confirmed that DPSCs could be induced into osteoblasts, lipoblasts and chondroblasts, indicating a multi‐lineage differentiation potential. Scale bar = 100 μm. (D) Transmission electron microscopy (TEM) images of DPSC‐IVs. Scale bar = 400 nm. (E) Western blot showed the presence of Alix, TSG101 and CD9 in both DPSCs and DPSC‐IVs, with absence of Calnexin as a negative marker in DPSC‐IVs. (F) Nanoparticle tracking analysis (NTA) showed the particle concentration and particle size of DPSC‐IVs.
**Figure S2:** Biocompatibility and biosafety of DPSC‐IVs. H&E staining showed no significant differences in the heart, liver, spleen, lung and kidney among different groups. Scale bar = 50 μm.

## Data Availability

The data that support the findings of this study are available from the corresponding author upon reasonable request.
