# Supplementary material for: Dental Pulp Stem Cell‐Derived Intracellular Vesicles Inhibit OSCC by Delivering PTEN to Suppress PI3K/AKT/mTOR Signalling Pathway
Source: Cell Prolif. 2026 Jun 10:e70248. Online ahead of print. doi: 10.1111/cpr.70248 (PMC13326028; doi:10.1111/cpr.70248)
Supplement: Supplementary file 1 — Figure S1: Characterisation of DPSC‐derived intracellular vesicles (DPSC‐IVs). (A) Schematic illustration of the DPSC‐IVs extraction by ultracentrifugation. (B) Images of DPSCs‐P0 and DPSCs‐P3 under light microscope. Scale bar = 200 μm. (C) Alizarin red, oil red and alizarin blue staining confirmed that DPSCs could be induced into osteoblasts, lipoblasts and chondroblasts, indicating a multi‐lineage differentiation potential. Scale bar = 100 μm. (D) Transmission electron microscopy (TEM) images of DPSC‐IVs. Scale bar = 400 nm. (E) Western blot showed the presence of Alix, TSG101 and CD9 in both DPSCs and DPSC‐IVs, with absence of Calnexin as a negative marker in DPSC‐IVs. (F) Nanoparticle tracking analysis (NTA) showed the particle concentration and particle size of DPSC‐IVs. Figure S2: Biocompatibility and biosafety of DPSC‐IVs. H&E staining showed no significant differences in the heart, liver, spleen, lung and kidney among different groups. Scale bar = 50 μm. [file CPR-9999-e70248-s001.docx]

**Supplementary Materials**

**Dental Pulp Stem Cell-Derived Intracellular Vesicles** **Inhibit OSCC by Delivering PTEN to Suppress PI3K/AKT/mTOR Signaling Pathway**

Yu Luo^1#^, Qiang Qin^1#^, Wenting She^1#^, Xiangying Wang^1^, Xiqin Li^1^, Chenxuan Shu^1^, Ruohan Li^1^, Ziwei Li^1^, Dongjie Fu^1*^, Yan He^2, 3*^ and Qingsong Ye^1, 3*^

1. Center of Regenerative Medicine and Department of Stomatology, Renmin Hospital of Wuhan University, Wuhan, China
2. Institute of Regenerative and Translational Medicine, Tianyou Hospital, Wuhan University of Science and Technology, Wuhan, China
3. Department of Oral and Maxillofacial Surgery, Massachusetts General Hospital, Harvard Medical School, Boston, MA, USA

* Corresponding authors.

Qingsong Ye

Center of Regenerative Medicine and Department of Stomatology, Renmin Hospital of Wuhan University, Wuhan, China. E-mail: qingsongye@whu.edu.cn

Yan He

Institute of Regenerative and Translational Medicine, Tianyou Hospital, Wuhan University of Science and Technology, Wuhan, China. E-mail: helen-1101@hotmail.com

Dongjie Fu

Center of Regenerative Medicine and Department of Stomatology, Renmin Hospital of Wuhan University, Wuhan, China. E-mail: dongjie_fu@163.com

^#^ These authors contributed equally to this work.

**Supplementary Materials**

**Figure S1. Characterization of DPSC-derived intracellular vesicles (DPSC-IVs).** (A) Schematic illustration of the DPSC-IVs extraction by ultracentrifugation. (B) Images of DPSCs-P0 and DPSCs-P3 under light microscope. Scale bar = 200 μm. (C) Alizarin red, oil red and alizarin blue staining confirmed that DPSCs could be induced into osteoblasts, lipoblasts and chondroblasts, indicating a multi-lineage differentiation potential. Scale bar = 100 μm. (D) Transmission electron microscopy (TEM) images of DPSC-IVs. Scale bar = 400 nm. (E) Western blot showed the presence of Alix, TSG101, and CD9 in both DPSCs and DPSC-IVs, with absence of Calnexin as a negative marker in DPSC-IVs. (F) Nanoparticle tracking analysis (NTA) showed the particle concentration and particle size of DPSC-IVs.

**Figure S2. Biocompatibility and biosafety of DPSC-IVs.** H&E staining showed no significant differences in the heart, liver, spleen, lung and kidney among different groups. Scale bar = 50 μm.
